# Structure and Function of PML Nuclear Bodies: A Brief Overview of Key Cellular Roles

**DOI:** 10.3390/biom15091291

**Published:** 2025-09-08

**Authors:** Karolina Dorosz, Lidia Majewska, Jacek Kijowski

**Affiliations:** 1 Biology Division, University of Chicago, Chicago, IL 60637, USA; kdorosz@uchicago.edu; 2 ESME Clinic, 30-548 Krakow, Poland; 3 Stem Cell Laboratory, Małopolska Centre of Biotechnology, Jagiellonian University, 31-007 Krakow, Poland; jacek.kijowski@uj.edu.pl

**Keywords:** promyelocytic leukemia nuclear bodies (PML-NBs), membrane-less organelles, SUMOylation, chromatin interaction, transcriptional regulation, protein quality control, antiviral response, acute promyelocytic leukemia

## Abstract

Promyelocytic leukemia nuclear bodies (PML-NBs) are dynamic membrane-less organelles (MLOs) located in the nucleus that serve as regulatory hubs for multiple cellular processes. This review examines current understanding of PML-NB structure, assembly mechanisms, and their diverse functional roles. We discuss how PML-NBs interact with chromatin to influence gene expression, regulate transcription factors, and participate in protein quality control. The review highlights their critical functions in tumor suppression, particularly in acute promyelocytic leukemia, and their role in intrinsic antiviral defense against various pathogens. Despite significant advances in the field, key questions remain regarding the mechanistic triggers of PML-NB formation and their common roles across different pathologies. Further elucidation of these aspects may provide valuable insights for developing therapeutic approaches targeting the PML-NB axis in disease treatment.

## 1. Introduction

PML-NBs are dynamic, evolutionarily conserved, nuclear MLOs exhibiting multivariate roles critical to maintaining cellular homeostasis [1,2]. Since the identification of the significant role that PML-NBs play in acute promyelocytic leukemia (APL), they have been extensively studied [1]. While PML-NBs are an inherent organelle of the nucleus in physiological conditions, their abundance tends to increase in response to cellular stress or immune signals, such as oxidative stress or interferon (IFN) signaling [1]. Concurrently, PML-NB dysfunctions have been associated with pathological conditions including, but not limited to, APL and C9orf72 amyotrophic lateral sclerosis (ALS) [1,3]. In addition to their critical role in endogenous pathologies, PML-NBs are also involved in innate immunity within antiviral responses by modulation of viral genomes [4].

Despite extensive research, significant knowledge gaps remain regarding the mechanistic formation of PML-NBs in response to cellular stressors and how their diverse context-dependent functions connect to various pathologies. The role of PML-NBs in maintaining and restoring cellular homeostasis is multifaceted and its full understanding has the potential to offer significant advances in the understanding and treatment of pathologies [4,5]. While substantial progress continues to be made, the multivalency of PML-NB functions as well as their cellular location, dynamic structure and size make research in this area difficult [5]. This review highlights the current state of knowledge on PML-NB structure, their role in endogenous translational regulation, and their involvement in antiviral response, while underscoring the outstanding challenges driving ongoing research efforts [6,7,8,9,10,11,12].

## 2. Structure and Assembly

**Figure 1:**
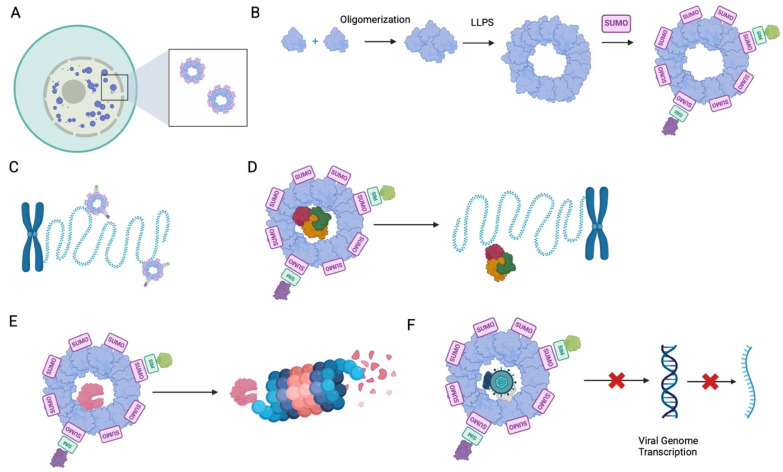
Graphical abstract of key cellular roles of PML-NBs. (**A**) Representative location of PML-NBs in the cell. (**B**) Simplified PML-NB assembly mechanism. (**C**) PML-NB–chromatin interactions. (**D**) P53 (red) chromatin interactions mediated by DAXX (yellow) and ATRX (green) at PML-NBs. (**E**) PML-NB-mediated targeting of DRiPs for proteasomal degradation. (**F**) PML-NB-mediated restriction of viral genome replication via virus enclosure. Created with BioRender.com.

The structural organization of PML-NBs is central to their function as dynamic nuclear compartments. These MLOs appear as spherical structures ranging from 0.1 to 2 µm in diameter and consist of an external protein scaffold surrounding a granular or heterogeneous interior (Figure 1A) [1]. Their primary structural component is the PML tumor suppressor protein, which serves as the scaffold for PML-NB assembly [1,6]. The PML gene encodes seven major protein isoforms (PML I–PML VIIb), six of which are nuclear (PML I–PML VI) and capable of forming PML-NBs [7]. These isoforms share a conserved N-terminal RBCC domain (RING, B-boxes, and coiled-coils), which is essential for oligomerization and self-assembly [7,8]. The RING domains are particularly critical, as they form tetramers promoting higher order polymer assembly contributing to PML-NB formation [9]. Recent evidence suggests that these interactions drive liquid–liquid phase separation (LLPS), enabling PML molecules to dynamically cluster into PML-NBs (Figure 1B) [7,8,9]. Consistently, RBCC domain mutations that impair oligomerization or disrupt LLPS prevent PML-NB formation in cellular models [8,9].

Beyond oligomerization, post-translational modifications (PTMs) play a critical role in stabilizing PML-NBs and recruiting partner proteins [13]. The PML protein undergoes SUMOylation at three key lysine residues (K65, K160, and K490) and contains a small-ubiquitin-like-modifier-(SUMO)-interacting motif (SIM), which facilitates non-covalent interactions with other SUMOylated proteins (Figure 1B). These proteins may include SP100, DAXX, or other client proteins, which only transiently pass through PML-NBs [1]. While SUMO–SIM interactions contribute to the structural maturation of PML-NBs, they are not strictly required for nucleation, as PML mutants lacking SUMOylation sites or SIMs can still form nuclear bodies [13]. However, these mutant PML-NBs fail to efficiently recruit interacting proteins, rendering them functionally impaired [13,14]. Given that PML-NBs act as regulatory hubs, their ability to sequester, modify, or degrade nuclear proteins depends heavily on SUMO-mediated interactions. Thus, while RBCC-driven oligomerization establishes the core scaffold of PML-NBs, SUMO–SIM interactions refine their structural integrity and functional versatility.

## 3. Chromatin Interactions

PML-NB–chromatin interactions are heavily context-dependent and influence chromatin organization, as well as gene regulation (Figure 1C). PML-NBs associate with chromatin in a highly dynamic, specific manner and may be transient or prolonged [15,16]. A key example of interaction specificity is the association of PML-NBs with the major histocompatibility complex (MHC) gene cluster, which contains genes encoding MHC I and II, such as the *DRA* gene [16,17]. This interaction has been found to persist regardless of transcriptional activity or the cell cycle phase [17]. Further, PML-NBs still associated with the gene cluster even after it was translocated from chromosome 6 onto chromosome 18 in the B-lymphoblastoid cell line [17]. What is more, IFN-ɣ, a known inducer of PML-NBs, has been shown to facilitate a prolonged PML-NB-DRA gene interaction that leads to epigenetic memory in HeLa cells [15,16]. PML-NBs have persisted in the vicinity of the DRA gene for up to 96 h post IFN-ɣ treatment and resulted in faster DRA transcription upon IFN-ɣ restimulation [16]. Further, PML-depleted cells did not experience memory upon IFN-ɣ treatment and restimulation, which suggests a central role of PML-NB in this phenomenon [16].

Alternatively, PML-NB association with active regulatory regions has also been recorded in multiple cellular settings [10,15,18]. In mouse embryonic fibroblasts, it has been observed that PML-NBs interact with specific, telomeric gene regions on the short arm of the Y chromosome [18]. PML-NBs contribute to the 3D chromatin organization and create foci free of the DNMT3A DNA methyltransferase, which maintains a hypomethylated chromatin state at the promoter CpG islands [18]. Interestingly, though oxidative stress resulted in an increased number of PML-NBs, their chromatin interactions decreased, yet gene expression remained unaffected [18]. Concurrently, studies also show that PML-NBs may associate with telomeres in cancer cells that rely on alternative lengthening of telomeres (ALT) for telomere maintenance [10]. In these cells, PML-NBs assemble at damaged telomere sites induced by stress to promote homology-directed repair [10]. However, it has been demonstrated that protein condensates continue to form at telomeres in ALT cells even in absence of PML, as long as SUMO-SIM interactions are maintained [11]. While these findings demonstrate the ability PML-NBs to form specific chromatin interactions, mechanisms driving this specificity require further research to fully be elucidated [10].

## 4. Transcriptional Regulation and Protein Quality Control

PML-NBs are actively involved in transcriptional regulation via protein interactions [19,20]. These interactions are multifaceted and may concern a variable array of proteins, thus implicating PML-NBs in modulation of multivalent cellular processes. For example, PML-NB interaction is critical for the transcriptional activity of the p53 tumor suppressor protein in thymocytes [19]. p53 is expressed under cellular stress and induces senescence followed by cell death to prevent tumorigenesis [20]. While p53 can be transcribed in PML-deficient thymocytes, its functionality is negatively impacted and transcripts of its target genes such as *p21* and *Bax*, hallmarks of cellular senescence and apoptosis, respectively, are less abundant [19]. Similarly, in U87-T glioblastoma cells, the DAXX and ATRX proteins, frequently found in PML-NBs, seem to mediate p53–chromatin binding via facilitating chromatin accessibility (Figure 1D) [20]. Within these cells, PML-NBs act as dynamic reservoirs that transiently encapsulate DAXX and ATRX, enabling their post-translational modifications, particularly SUMOylation [21]. Upon cellular stress signals, these modified proteins are released from PML-NBs and recruited to chromatin sites where p53 binding is required [20]. At these sites, DAXX and ATRX facilitate chromatin remodeling, creating an accessible landscape that enhances p53 recruitment and subsequent transcriptional activation of target genes [20]. This controlled release and deployment mechanism ensures precise spatial and temporal regulation of p53-dependent transcription [20]. However, the TNF-related apoptosis-inducing ligand receptor 2 (TRAIL-R2), which facilitates p53 degradation, also interacts with p53 at PML-NBs [22]. Further, PML-NBs are indispensable to this interaction between TRAIL-R2 and p53, as it does not occur in PML-depleted cells [22]. These contrasting findings highlight the multivariate and context-dependent role of PML-NBs in transcriptional regulation.

The Aire transcription factor serves as another example of the central role of PML-NBs in transcription regulation. Aire is a critical transcription factor responsible for the development of T cell central tolerance and its dysfunction causes severe autoimmune complications [23]. Some disease-causing Aire mutants have been found to accumulate in PML-NBs [12]. Further, a subset of these mutants has been shown to undergo SUMOylation at PML-NBs, likely as a means of targeting them for proteasomal degradation [12]. Interestingly, PML-NBs have been found to function as stress-induced overflow storage compartments for defective ribosomal products (DRiPs) prior to proteasomal degradation, which could explain why defective Aire mutants localize to PML-NBs (Figure 1E) [12,24]. What is more, dysfunction of DRiP degradation mechanisms causes solidification of PLM-NBs, which impairs cell homeostasis and survival [24]. Recently, impaired PML-NB assembly and dysfunctions of DRiP handling mechanisms have been identified as a potential hallmark of C9orf72 ALS [3]. The association of PML-NB dysfunction with ALS is a significant discovery; however, more research is required to fully understand the role that these structures may play in the ALS pathology.

## 5. Antiviral Response

PML-NBs are known to modulate viral genomes and play an important role in intrinsic antiviral response [25]. A key mechanism of PML-NB antiviral activity involves direct viral gene repression. PML-NBs can sequester viral genomes and facilitate their modifications, which repress transcription (Figure 1F). For example, herpes simplex virus 1 (HSV1), member of the Herpesviridae family, known for its prolonged latency periods following initial infection, is regulated by PML-NBs [26,27]. The quiescent HSV1 genome localizes to the PML-NBs where its latency-associated transcript (LAT) expression is restricted [26,28]. It has been demonstrated that PML-NB-enclosed HSV1 acquires H3K9me3 modifications associated with transcriptional repression [27]. These modifications are primarily performed by the methyltransferase SETDB1, zinc finger protein MORC2 and the human silencing hub complex (HUSH). Interestingly, these molecular actors maintain HSV-1 silencing in PML-deficient cells, while LAT repression seems to be dependent on the presence of PML, suggesting a complex silencing mechanism [26,27]. The central role of PML-NBs in HSV1 genome regulation is also highlighted by HSV1′s own defense mechanisms against host immunity, which target PML [25]. HSV1 E3 ubiquitin ligase ICP0 specifically targets SUMOylated PML protein for proteasomal degradation to restore active infection [29] Alternatively, the stress hormone, cortisol, has been shown to mediate PML degradation via autophagy, which also restores viral genome transcription [30].

Another group of viruses targeted by PML-NBs are the Adenoviruses (AdVs) [31]. The AdV genome transcription is severely impacted by PML-NBs in the IFN-induced innate antiviral response. In fact, it has been found that IFN-ɑ/IFN-ɣ stimulation itself is insufficient to decrease viral transcription when PML is depleted [32]. Specifically, the DAXX PML-NB protein has been identified as critical for restricting AdV replication via its interactions with the E4 ORF3 AdV protein, which disrupts the focalized spherical structure of PML-NBs [32]. Concurrently, to prevent DAXX activity, the AdV E1B-55K protein targets DAXX for proteasomal degradation, in a mechanism reminiscent of that of ICP0 in HSV1 [29,33]. While these examples demonstrate significant advancements in the understanding of PML-NBs’ antiviral activity, many PML-NB interactions with the viral genome remain not understood and require further research efforts (see Table 1).

## 6. Conclusions

Promyelocytic leukemia nuclear bodies (PML-NBs) are dynamic, membrane-less organelles central to numerous regulatory cellular processes. Their structure—built on the PML protein scaffold through liquid–liquid phase separation—facilitates diverse functions ranging from chromatin interactions and epigenetic regulation to transcriptional control and antiviral defense. Their ability to mediate SUMOylation, sequester and release client proteins such as p53, and participate in protein quality control underscores their multifunctional importance in maintaining cellular homeostasis. PML-NBs have also emerged as critical players in disease contexts, particularly in cancer, neurodegeneration, and viral infections.

## 7. Future Directions

While substantial insights into PML-NB biology have been gained, several key questions remain. For instance, the mechanistic triggers by which oxidative stress and immune signaling initiate PML-NB nucleation are not yet fully understood. The multifaceted and sometimes contradictory roles of PML-NBs in transcription regulation—such as both promoting and inhibiting p53 activity—warrant further systems-level studies to elucidate their context-dependent behavior. Similarly, their role in defective ribosomal product (DRiP) handling and the consequences of impaired PML-NB proteostasis remain underexplored and could offer insights into pathologies like ALS. The antiviral activities of PML-NBs—particularly their role in silencing viral genomes independent of cytokine stimulation—should be investigated more deeply, including how viruses evolve mechanisms to disrupt PML-NB function. Future research should focus on identifying shared pathways that converge on PML-NBs during stress responses, infection, and tumorigenesis. Answering these questions may uncover conserved molecular mechanisms and therapeutic targets linked to PML-NB biology.

## Figures and Tables

**Table 1:** Overview of key PML-NB-interacting proteins and their primary functions, interaction mechanisms, and disease relevance.

Protein(s)	Primary Function	Interaction with PML-NBs	Disease Relevance	Reference(s)
SP100	Transcriptional regulation, chromatin remodeling	Core component; recruited via SUMO-SIM interactions	Autoimmune disorders; Viral defense	Lallemand-Breitenbach & de Thé, 2010 [1]
DAXX	Transcriptional co-repressor; H3.3 chaperone	Dynamically associates with PML-NBs; undergoes SUMOylation within PML-NBs	Cancer; Viral defense; Neurodegeneration	Gulve et al., 2022 [20]; Schreiner et al., 2010 [33]
ATRX	Chromatin remodeling	Forms complex with DAXX; facilitates p53 chromatin binding	ALT cancers; X-linked α-thalassemia/mental retardation syndrome	Gulve et al., 2022 [20]; Yu & Zhang, 2025 [10]
p53	Tumor suppressor; regulates cell cycle arrest and apoptosis	Activity modulated within PML-NBs	Cancer; Cellular senescence	Guo et al., 2000 [19]; Wang et al., 2023 [21]
TRAIL-R2	Death receptor	Interaction with p53 occurs at PML-NBs	Cancer; Apoptosis resistance	Willms et al., 2021 [22]
Aire	Transcription factor for central tolerance	Disease-causing mutants accumulate in PML-NBs	Autoimmune polyendocrinopathy-candidiasis-ectodermal dystrophy (APECED)	Huoh et al., 2020 [12]; Peterson et al., 2008 [23]
SUMO1/2/3	Post-translational modifier	Essential for PML-NB maturation and protein recruitment	Multiple (cancer, viral defense, neurodegeneration)	Sahin et al., 2014 [13]; Sloan et al., 2015 [25]
RNF4	SUMO-targeted ubiquitin ligase	Regulates PML-NB turnover	APL treatment response	Lallemand-Breitenbach & de Thé, 2010 [1]
SETDB1	Histone methyltransferase	Mediates H3K9me3 at viral genomes within PML-NBs	Viral latency	Roubille et al., 2024 [27]
MORC2	ATPase; chromatin remodeling	Component of HUSH complex at PML-NBs	Viral latency; Charcot–Marie–Tooth disease	Roubille et al., 2024 [27]

## Data Availability

No new data were created or analyzed in this study.

## References

[1] Lallemand-Breitenbach V., de Thé H. (2010). PML nuclear bodies. Cold Spring Harb. Perspect. Biol..

[2] Borden K.L. (2008). Pondering the puzzle of PML (promyelocytic leukemia) nuclear bodies: Can we fit the pieces together using an RNA regulon?. Biochim. Biophys. Acta.

[3] Goswami A., Carra S. (2024). PML nuclear bodies: New players in familial amyotrophic lateral sclerosis-frontotemporal dementia?. Neural Regen. Res..

[4] Ryabchenko B., Šroller V., Horníková L., Lovtsov A., Forstová J., Huérfano S. (2023). The interactions between PML nuclear bodies and small and medium size DNA viruses. Virol. J..

[5] Corpet A., Kleijwegt C., Roubille S., Juillard F., Jacquet K., Texier P., Lomonte P. (2020). PML nuclear bodies and chromatin dynamics: Catch me if you can!. Nucleic Acids Res..

[6] Shen T.H., Lin H.K., Scaglioni P.P., Yung T.M., Pandolfi P.P. (2006). The mechanisms of PML-nuclear body formation. Mol. Cell.

[7] Nisole S., Maroui M.A., Mascle X.H., Aubry M., Chelbi-Alix M.K. (2013). Differential Roles of PML Isoforms. Front. Oncol..

[8] Wu W., Tan Y., Yin H., Jiang M., Jiang Y., Ma X., Yin T., Li Y., Zhang H., Cai X. (2023). Phase separation is required for PML nuclear body biogenesis and function. FASEB J..

[9] Wang P., Benhenda S., Wu H., Lallemand-Breitenbach V., Zhen T., Jollivet F., Peres L., Li Y., Chen S.J., Chen Z. (2018). RING tetramerization is required for nuclear body biogenesis and PML sumoylation. Nat. Commun..

[10] Yu X., Zhang H.L. (2025). Biomolecular Condensates in Telomere Maintenance of ALT Cancer Cells. J. Mol. Biol..

[11] Zhao R., Xu M., Yu X., Wondisford A.R., Lackner R.M., Salsman J., Dellaire G., Chenoweth D.M., O’Sullivan R.J., Zhao X. (2024). SUMO promotes DNA repair protein collaboration to support alternative telomere lengthening in the absence of PML. Genes Dev..

[12] Huoh Y.S., Wu B., Park S., Yang D., Bansal K., Greenwald E., Wong W.P., Mathis D., Hur S. (2020). Dual functions of Aire CARD multimerization in the transcriptional regulation of T cell tolerance. Nat. Commun..

[13] Sahin U., Ferhi O., Jeanne M., Benhenda S., Berthier C., Jollivet F., Niwa-Kawakita M., Faklaris O., Setterblad N., de Thé H. (2014). Oxidative stress-induced assembly of PML nuclear bodies controls sumoylation of partner proteins. J. Cell Biol..

[14] Banani S.F., Rice A.M., Peeples W.B., Lin Y., Jain S., Parker R., Rosen M.K. (2016). Compositional Control of Phase-Separated Cellular Bodies. Cell.

[15] Kiesslich A., von Mikecz A., Hemmerich P. (2002). Cell cycle-dependent association of PML bodies with sites of active transcription in nuclei of mammalian cells. J. Struct. Biol..

[16] Gialitakis M., Arampatzi P., Makatounakis T., Papamatheakis J. (2010). Gamma interferon-dependent transcriptional memory via relocalization of a gene locus to PML nuclear bodies. Mol. Cell. Biol..

[17] Shiels C., Islam S.A., Vatcheva R., Sasieni P., Sternberg M.J., Freemont P.S., Sheer D. (2001). PML bodies associate specifically with the MHC gene cluster in interphase nuclei. J. Cell Sci..

[18] Kurihara M., Kato K., Sanbo C., Shigenobu S., Ohkawa Y., Fuchigami T., Miyanari Y. (2020). Genomic Profiling by ALaP-Seq Reveals Transcriptional Regulation by PML Bodies through DNMT3A Exclusion. Mol. Cell.

[19] Guo A., Salomoni P., Luo J., Shih A., Zhong S., Gu W., Pandolfi P.P. (2000). The function of PML in p53 dependent apoptosis. Nat. Cell Biol..

[20] Gulve N., Su C., Deng Z., Soldan S.S., Vladimirova O., Wickramasinghe J., Zheng H., Kossenkov A.V., Lieberman P.M. (2022). DAXX-ATRX regulation of p53 chromatin binding and DNA damage response. Nat. Commun..

[21] Wang H., Guo M., Wei H., Chen Y. (2023). Targeting p53 pathways: Mechanisms, structures, and advances in therapy. Signal Transduct. Target. Ther..

[22] Willms A., Schupp H., Poelker M., Adawy A., Debus J.F., Hartwig T., Krichel T., Fritsch J., Singh S., Walczak H. (2021). TRAIL-receptor 2-a novel negative regulator of p53. Cell Death Dis..

[23] Peterson P., Org T., Rebane A. (2008). Transcriptional regulation by AIRE: Molecular mechanisms of central tolerance. Nat. Rev. Immunol..

[24] Mediani L., Guillén-Boixet J., Vinet J., Franzmann T.M., Bigi I., Mateju D., Carrà A.D., Morelli F.F., Tiago T., Poser I. (2019). Defective ribosomal products challenge nuclear function by impairing nuclear condensate dynamics and immobilizing ubiquitin. EMBO J..

[25] Sloan E., Tatham M.H., Groslambert M., Glass M., Orr A., Hay R.T., Everett R.D. (2015). Analysis of the SUMO2 Proteome during HSV-1 Infection. PLoS Pathog..

[26] Catez F., Picard C., Held K., Gross S., Rousseau A., Theil D., Sawtell N., Labetoulle M., Lomonte P. (2012). HSV-1 genome subnuclear positioning and associations with host-cell PML-NBs and centromeres regulate LAT locus transcription during latency in neurons. PLoS Pathog..

[27] Roubille S., Escure T., Juillard F., Corpet A., Néplaz R., Binda O., Seurre C., Gonin M., Bloor S., Cohen C. (2024). The HUSH epigenetic repressor complex silences PML nuclear body-associated HSV-1 quiescent genomes. Proc. Natl. Acad. Sci. USA.

[28] Alandijany T., Roberts A.P.E., Conn K.L., Loney C., McFarlane S., Orr A., Boutell C. (2018). Distinct temporal roles for the promyelocytic leukaemia (PML) protein in the sequential regulation of intracellular host immunity to HSV-1 infection. PLoS Pathog..

[29] Ip W.-H., Tatham M.H., Krohne S., Gruhne J., Melling M., Meyer T., Gornott B., Bertzbach L.D., Hay R.T., Rodriguez E. (2023). Adenovirus E1B-55K controls SUMO-dependent degradation of antiviral cellular restriction factors. J. Virol..

[30] Li W., Luo Z., Yan C.Y., Wang X.H., He Z.J., Ouyang S.H., Yan C., Liu L.F., Zhou Q.Q., Mu H.L. (2020). Autophagic degradation of PML promotes susceptibility to HSV-1 by stress-induced corticosterone. Theranostics.

[31] Hofmann S., Stubbe M., Mai J., Schreiner S. (2021). Double-edged role of PML nuclear bodies during human adenovirus infection. Virus Res..

[32] Ullman A.J., Hearing P. (2008). Cellular proteins PML and Daxx mediate an innate antiviral defense antagonized by the adenovirus E4 ORF3 protein. J. Virol..

[33] Schreiner S., Wimmer P., Sirma H., Everett R.D., Blanchette P., Groitl P., Dobner T. (2010). Proteasome-dependent degradation of Daxx by the viral E1B-55K protein in human adenovirus-infected cells. J. Virol..

